# Predictors of tuberculosis (TB) and antiretroviral (ARV) medication non-adherence in public primary care patients in South Africa: a cross sectional study

**DOI:** 10.1186/1471-2458-13-396

**Published:** 2013-04-26

**Authors:** Pamela Naidoo, Karl Peltzer, Julia Louw, Gladys Matseke, Gugu Mchunu, Bomkazi Tutshana

**Affiliations:** 1 Population Health, Health Systems and Innovation (PHHSI)/HIV/STIs and TB (HAST) Research Programmes, Human Sciences Research Council, Pretoria, Durban and Cape Town, South Africa, Private Bag X 9182, Cape Town 8000, South Africa; 2 Department of Psychology, University of the Western Cape, Cape Town, South Africa; 3 Department of Psychology, University of Limpopo, Turfloop, South Africa; 4 ASEAN, Institute for Health Development, Mahidol University, Salaya, Thailand; 5 Department of Nursing, University of KwaZulu Natal, Durban, South Africa

**Keywords:** Adult TB patients, Bio-Psycho-Social factors, Anti-TB treatment, ART, High burden country, Adherence to anti-TB treatment and ART

## Abstract

**Background:**

Despite the downward trend in the absolute number of tuberculosis (TB) cases since 2006 and the fall in the incidence rates since 2001, the burden of disease caused by TB remains a global health challenge. The co-infection between TB and HIV adds to this disease burden. TB is completely curable through the intake of a strict anti-TB drug treatment regimen which requires an extremely high and consistent level of adherence.The aim of this study was to investigate factors associated with adherence to anti-TB and HIV treatment drugs.

**Methods:**

A cross-sectional survey method was used. Three study districts (14 primary health care facilities in each) were selected on the basis of the highest TB caseload per clinic. All new TB and new TB retreatment patients were consecutively screened within one month of anti-tuberculosis treatment. The sample comprised of 3107 TB patients who had been on treatment for at least three weeks and a sub-sample of the total sample were on both anti-TB treatment and anti-retro-viral therapy(ART) (N = 757). Data collection tools included: a Socio-Demographic Questionnaire; a Post-Traumatic-Stress-Disorder (PTSD) Screen; a Psychological Distress Scale; the Alcohol Use Disorder Identification Test (AUDIT); and self-report measures of tobacco use, perceived health status and adherence to anti-TB drugs and ART.

**Results:**

The majority of the participants (N = 3107) were new TB cases with a 55.9% HIV co-infection rate in this adult male and female sample 18 years and older. Significant predictors of non-adherence common to both anti-TB drugs and to dual therapy (ART and anti-TB drugs) included poverty, having one or more co-morbid health condition, being a high risk for alcohol mis-use and a partner who is HIV positive. An additional predictor for non-adherence to anti-TB drugs was tobacco use.

**Conclusions:**

A comprehensive treatment programme addressing poverty, alcohol mis-use, tobacco use and psycho-social counseling is indicated for TB patients (with and without HIV). The treatment care package needs to involve not only the health sector but other relevant government sectors, such as social development.

## Background

Despite the downward trend in the absolute number of TB cases since 2006 and the fall in the incidence rates since 2001, the burden of tuberculosis (TB) disease remains a global health challenge [1]. TB is completely curable through the intake of a strict drug treatment regimen. The Directly Observed Treatments Short-Course Strategy (DOTS) introduced by the World Health Organization (WHO) and subsequently the Stop TB Strategy is an inexpensive strategy that could prevent millions of TB cases and deaths. TB/HIV co-infection and multi-drug resistant (MDR)/ extensive drug-resistant (XDR)TB are given focal attention in the Stop TB Strategy and one of its primary TB targets is to reduce by half the TB prevalence rates by 2015 relative to 1990 [1].

The HIV and AIDS pandemics have exacerbated the public health dimensions of TB due to the fact that many individuals are co-infected. TB is the leading cause of death among people who are HIV positive. In the African region, which accounted for 82% of the new TB cases that were also HIV positive, an estimated 900 000 (39%) of the 2.3 million people who developed TB were living with HIV [1].

### Adherence to anti-TB treatment and to treatment for HIV/TB co-infection

Poor adherence to the prescribed anti-TB treatment programmes, such as those falling under the DOTS strategy, is one of the factors associated with low cure rates for TB. In addition, inconsistent adherence to the anti-TB drug regimen may lead to multiple and extensive drug resistance (MDR-TB and XDR-TB respectively) making it difficult to achieve high cure rates. In individuals with TB/HIV dual infection receiving treatment, correct adherence to anti-TB drugs and anti-retroviral therapy (ART) are essential for good treatment outcomes [2]. Poor and inconsistent adherence to ART can also lead to drug resistance and even death, an outcome which is similar to non-adherence to anti-TB drugs [2,3].

The categories of factors influencing adherence to drug treatments for most health-related conditions, include: practitioner’s negative assumptions about their patients; psychological attributes of the patient; environmental, social and cultural factors; treatment characteristics; and the doctor–patient relationship [3,4]. Many quantitative and qualitative studies conducted in South Africa (SA) have identified factors known to influence adherence to anti-TB treatment [5-7]. Qualitative studies exploring adult TB patient’s adherence to anti-TB treatment at public health sites in SA found that the factors that influenced patient co-operation included: social and economic resources; causal attributions assigned to TB; the social, cultural, economic, disease-related and psychological challenges faced as a consequence of having TB; quality of health care received; use of the traditional healing system; and the patient’s HIV status [5,7]. Quantitative studies examining the factors that influence adherence to anti-TB treatment regimens found the following to be important: a good patient–practitioner relationship, ability of the patient to disclose medication use to members of their social network and regular clinic visits [8,9].

Numerous studies have also been conducted in Africa and globally on factors influencing adherence to anti-TB treatment and adherence to the dual treatment approach for TB and HIV [2,10,11]. A systematic review of qualitative research, exploring patient adherence to tuberculosis treatment, conducted by Munro et al found that the following four factors interact to affect adherence to TB treatment: structural factors, including poverty and gender discrimination; the social context; health service factors and personal factors [5]. An African-based qualitative study which explored barriers and facilitators of adherence to anti-TB treatment and concomitant TB and HIV treatment in Ethiopia, found that adherence to TB treatment was positively influenced by beliefs in the curability of TB, beliefs in the severity of TB in the presence of HIV infection and support from family members and health professionals [11].

A systematic review done on studies conducted in the US and Canada on adherence to treatment for latent tuberculosis infection found that there was a “sub-optimal” level of adherence indicated across all studies [12]. The factors influencing adherence were clinic facilities, treatment characteristics and patient factors although the association between adherence and these factors was found to be inconsistent [12]. Adherence to ARVs as compared to anti-TB drugs is, however, reported to be high the world over and Corless et al based a quantitative study conducted in clinics in Durban, SA, on this premise [10]. They found that in fact adherence to ARVs in their study was also high [10]. Amuha et al found that the most important factor associated with non-adherence to anti-TB medication in individuals co-infected with HIV was being on a continuous treatment regimen phase as compared to the intensive treatment phase. Confounding factors influencing the association between the stage of the TB regimen and non-adherence were alcohol consumption, being on ARVs and smoking [13]. The exact nature of these confounding factors and its influence on non-adherence has not been interrogated.

Relatively few studies have looked specifically at the sub-group of individuals on anti-TB treatment who also meet the criteria for alcohol use disorders in South Africa (SA). This study forms part of a larger study entitled: Screening and brief interventions for hazardous and harmful alcohol use among patients with active tuberculosis attending primary public care clinics in SA. There is adequate evidence in the international literature to confirm that TB medication non-adherence is associated with the use and mis-use of alcohol [14,15]. In SA, however, the association between alcohol (mis)use and non-adherence to anti-TB treatment and dual therapy in those individuals co-infected with HIV has been not been adequately studied. In addition, the way in which individual mental health risk factors, such as distress and post-traumatic stress, mediate TB and TB/HIV health outcomes for individuals mis-using alcohol, has been neglected.

The value of this study is that it used a different methodology to most other TB treatment adherence studies. The study uses different questionnaires (such as the measures of distress and PTSD) and different outcome measures (namely, adherence to anti-TB treatment, and adherence to ART and anti TB treatment (dual therapy) as compared to other adherence studies. Consequently, the primary aim of this study, using base-line data was to investigate the factors that are associated with non-adherence to anti-TB drugs for those with active TB and to ART and anti-TB drugs taken by individuals who have dual infection. The secondary aim, which also addresses a gap in research, was to specifically investigate the significance of alcohol misuse as a predictive factor for non-adherence to anti-TB drugs and to dual therapy (namely, anti-TB drugs and ART).

## Methods

### Study design, sample and procedure

This study is a cross-sectional survey. Fourteen (14) public primary health care clinics in only one district in each of three provinces, namely, Northern Cape, Eastern Cape and Kwa-Zulu Natal in SA with the highest TB caseload were included in the study. All new TB and new retreatment patients were consecutively screened within one month of anti-TB treatment. The public primary health care clinics that were utilized in this study followed the SA Department of Health’s (DoH) Guidelines for TB treatment who in turn are guided by the WHOs TB treatment guidelines framed by the Stop TB strategy which strongly recommends the DOT programme [16]. The DOT programme requires intensive involvement and monitoring by the clinic staff but the treatment success is also dependent on the patient adhering strictly to the treatment guidelines. In this study a total of 3129 potential participants were screened and 22 (0.7%) refused to participate. It was not necessary to perform an attrition analysis due to the good response rate. In the data analysis the total sample size used was 3107 participants (N = 3107). All the participants’ in this study were taking anti-TB treatment for at least three weeks, which fell into the intensive phase of the DOTS programme which required them to attend the clinic during the week and take their anti-TB drugs dose at their homes over the week-end.

Within the larger sample there was a sub-sample of patients who were HIV positive (N = 1729) and a proportion of the HIV positive patients (44%) who were on ART (N = 757).

A screening interview was conducted by trained research assistants over a period of six (6) months in 2011. A health care provider who identified a new TB treatment or retreatment patient 18 years and above informed the patient about the study and referred the patient for participation if they were willing. A consenting procedure was adopted prior to the start of the screening interview. Ethical approval was received from the Human Sciences Research Council Research Ethics Committee (Protocol REC No.1/16/02/11) and the Department of Health (DoH) in SA.

### Data collection tools

The data collection tools specified in this section was administered to all the participants in this study.

#### Socio-demographic questionnaire

A researcher-designed questionnaire was used to record information on participants’ age, gender, educational level, marital status, income, employment status, dwelling characteristics and residential status. Using a previously designed questionnaire, which is also extensively used in the Human Sciences Research Council (HSRC_SA) national health surveys, *poverty* was assessed by five (5) pertinent items on the questionnaire by asking about the availability or non-availability of shelter, fuel or electricity, clean water, food and cash income in the past week [17]. Response options ranged from 1 = “Not one day” to 4 = “Every day of the week”. Participants ranked high on poverty if they had higher scores on non-availability of essential items. The total poverty score ranged from 5 to 20 with the following categories: 5 indicating low poverty; 6 to 12 indicating a medium level of poverty; and 13 to 20 indicating high levels of poverty. The Cronbach alpha for the poverty index in this study was fairly good (0.89).

#### The Kessler Psychological Distress Scale (K-10)

The Kessler Psychological Distress Scale (K-10) was used to measure global psychological distress [18,19]. The K-10 measures the following symptoms over the preceding 30 days: nervousness, hopelessness, restlessness, depression, worthlessness and tiredness. The frequency with which each of these items was experienced was recorded using a five-point Likert scale ranging from “none of the time” to “all the time”. Increasing total scores reflect an increasing degree of psychological distress. The K-10 has been shown to capture variability related to non-specific depression, anxiety and substance abuse [19]. This scale serves to identify individuals who are likely to meet formal definitions for anxiety and/or depressive disorders, as well as to identify individuals with sub-clinical illness who may not meet formal definitions for a specific disorder [18]. This scale is increasingly used in population based mental health research and has been validated in multiple settings [20,21] including HIV positive individuals in SA [22]. The internal reliability coefficient for the K-10 in this study was alpha = 0.92 which is fairly high.

#### Alcohol consumption

The 10-item Alcohol Use Disorder Identification Test (AUDIT) [23] assesses alcohol consumption level, symptoms of alcohol dependence and problems associated with alcohol use. The AUDIT largely measures heavy episodic drinking with only one (1) item measuring binge drinking. Heavy episodic drinking is defined as the consumption of six standard drinks or more on a single occasion. A standard drink in this instance is 10 g of alcohol. In SA a standard drink is 12 g of alcohol and not 10 g. The AUDIT is reported to be less sensitive at identifying risk drinking in women [24] so it was recommended that the cut-off point for the binge drinking measure is reduced by one (1) unit among women in SA and the overall heavy episodic drinking measure is also reduced due to the fact that a standard drink is 12 g of alcohol (and not 10 g as stipulated by the AUDIT). Responses to items on the AUDIT are rated on a four-point-Likert scale from 0 to 4, for a maximum score of 40 points. A cut off score of 8 indicates a tendency to problematic drinking. The AUDIT was developed by the WHO as an effective screening instrument for alcohol use problems among patients seeking primary care for other medical problems in international settings including African countries (Kenya and Zimbabwe) [24-27]. The AUDIT has been validated in HIV patients in SA showing excellent sensitivity and specificity in detecting alcohol dependence and alcohol abuse as defined on the Mini-International Neuropsychiatric Interview (MINI) [26]. The MINI is an internationally recognized diagnostic tool in the form of a psychiatric interview.

The MINI used in a study of TB and HIV patients in primary care in Zambia also demonstrated good discriminatory ability in detecting MINI-defined current Alcohol Use Disorders (AUDs) (AUDIT = 0.98 for women and 0.75 for men) [27]. Cronbach alpha for the AUDIT in this sample was 0.92, indicating excellent reliability.

#### Additional self-report measures

(a) Tobacco use: Two researcher generated questions were asked about the use of tobacco products. The first question asked about current tobacco use and the second question asked about the frequency of use over the past month.

(b) Non-adherence to anti-TB treatment, non-adherence to ART, and HIV testing: Adherence was assessed by self-report. Whether a participant tested for HIV infection was also assessed by self-report. Anti-TB medication adherence was assessed with the following question: “In your tuberculosis treatment in the past 3–4 weeks how many percent (%) of your anti-tuberculosis medication did you take?” TB medication non-adherence was defined as having taken less than 90% of the anti-TB drugs. Self-reporting adherence behaviour over the past 3–4 week period for anti-TB drugs was asked due to the fact that the participants in this study were recruited only if they had started their TB treatment at least three weeks before being enrolled into the study.

 ART adherence was assessed with the question: “How many percent of your HIV medication did you take in the past 4 weeks?” ART non-adherence was defined as having taken less than 90% of ART. In each instance, that is for both ART and anti-TB treatment non-adherence, participants were required to mark the percentage adherence on an illustrated scale indicating progression from 0% to 100% adherence.

(c) Co-morbidity with other chronic medical conditions including hypertension, diabetes, depression, stomach ulcer, migraine headache, cancer, arthritis, asthma, diabetes, cholesterol were also ascertained.

### Data analysis

Data were analyzed using the Statistical Package for the Social Sciences (SPSS-version 19). Frequencies, means, and standard deviations, were calculated to describe the sample. Data were checked for normality distribution and outliers. For non-normal distribution non-parametric tests were used. Associations between TB medication and ART non-adherence were identified using logistics regression analyses. Following each univariate regression, multivariable logistic regression models were constructed. A total of 574 participants were used in the multivariate analysis. Independent variables from the univariate analyses were entered into the multivariable model if significant at P < 0.05 level. For each model, the R^2^ are presented to describe the amount of variance explained by the multivariable model. Probability below 0.05 was regarded as statistically significant.

## Results

### Characteristics of the final sample

Table 1 indicates that the sample included 671 (21.8%; N = 3107) retreatment cases, 2408 (78.2%; N = 3107) new TB cases and 55.6% HIV infected cases. A little below half the sample was 35 years and older. Half the sample reported medium levels of poverty, about a third (35.3%) good perceived health status with more participants (46.1%) reporting poor perceived health status. Other characteristics of significance include the following: 776 (25%) met the criteria for severe psychological distress and 16.4% for the category of medium risk for alcohol misuse on the AUDIT. Finally, 26.1% were non-adherent to the anti-TB treatment (min = 0, max = 100, mean = 77.0, median = 90.0, SD = 43.9).

**Table 1:** Characteristics of the total sample of TB patients and sub-sample of TB patients on ART

**Socio-demographics**	**Total TB patients (N = 3107)**	**Total TB-ART patients (N = 757)**
	**N (%)**	**N (%)**
Age 18–24	416 (13.5)	65 (8.7)
Age 25–34	1160 (37.7)	283 (37.8)
Age 35–44	861 (28.0)	252 (33.7)
Age 45 and older	639 (20.8)	148 (19.8)
Missing N	31	9
Female	1427 (46.5)	387 (51.7)
Male	1639 (53.5)	362 (48.3)
Missing N	41	8
Grade 7 or less	774 (25.2)	234 (31.5)
Grade 8–11	1415 (16.1)	330 (44.4)
Grade 12 or more	881 (28.7)	179 (24.1)
Missing N	37	14
Poverty low	1052 (35.5)	173 (24.4)
Poverty medium	1484 (50.1)	377 (53.1)
Poverty high	427 (14.4)	160 (22.5)
Missing N	144	47
Health variables		
Perceived health status		
Excellent/Very good	573 (18.6)	217 (28.8)
Good	1089 (35.3)	231 (30.2)
Fair/Poor	1423 (46.1)	305 (40.5)
Unknown N	22	4
TB retreatment patient	671 (21.8)	224 (30.0)
New TB patient	2408 (78.2)	523 (70.0)
Unknown N	28	10
HIV positive	1729 (55.9)	
HIV negative	1073 (34.5)	
HIV unknown status	305 (9.8)	
Chronic conditions		
Zero	1983 (72.5)	422 (65.7)
One	443 (16.2)	112 (17.4)
Two	194 (7.1)	60 (9.3)
Three or more	117 (4.3)	48 (7.5)
Unknown N	370	115
Severe psychological distress (K ≥ 30)	776 (25.0)	208 (27.5)
Alcohol: low risk (AUDIT 0–7)	2392 (78.0)	599 (80.0)
Medium (AUDIT 8–19)	504 (16.4)	119 (15.9)
High (AUDIT 20–40)	172 (5.6)	31 (4.1)
Unknown N	39	8
Current tobacco use	799 (26.2)	203 (27.4)
Partner HIV negative/unknown	2061 (71.6)	436 (61.8)
Partner HIV positive	818 (28.4)	269 (38.2)
Unknown/missing N	228 (7.3)	52 (6.9)
Sex partner on ART	300 (11.8)	355 (48.8)
TB non-adherence	812 (26.1)	315 (41.6)
ART non adherence		321 (42.4)

A sub-sample (N = 757) of the total sample was on dual anti-TB treatment and ART. The characteristics of this group were as follows: the majority of participants were between the ages of 25 and 44 years, half the sample reported medium levels of poverty, two-fifths (40.5%) reported poor perceived health status, 15.9% were at the medium risk category for alcohol misuse on the AUDIT, 38.2% had a partner who was HIV positive, and 42.4% were non-adherent to ART (min = 0, max = 100, mean = 64.8, median = 100.0, SD = 43.9). Of a total of 268 participants 83.8% were not adherent to both anti-TB and ART medication (r = 0.71).

### Predictors of non-adherence to TB treatment

Univariate analysis (see Table 2) shows that the following factors were more likely to be associated with non-adherence to TB treatment: being male [OR: 1.26 (1.07–1.48), p < 0.05], medium poverty [OR: 1.97 (1.62–2.41), p < 0.001], high levels of poverty [OR: 4.01 (3.12–5.10), p < 0.001], having one (1) chronic condition [OR: 1.54 (1.23–1.93), p < 0.001], having two (2) chronic conditions [OR: 1.84 (1.34–2.51), p < 0.001], having three or more chronic conditions [OR: 1.98 (1.34–2.92), p < 0.001], severe psychological distress [OR: 1.31 (1.09–1.57), p < 0.01], medium risk for alcohol misuse [OR: 1.74 (1.42–2.19), p < 0.001], high risk for alcohol misuse [OR: 2.42 (1.76–3.32), p < 0.001], tobacco use [OR: 2.09 (1.76–2.49), p < 0.001] and having a partner who is HIV positive [OR: 1.43 (1.10–1.84), p < 0.01].

**Table 2:** Predictors of non-adherence to TB medication (N = 3107)

**Socio-demographics**	**Crude OR (95% CI)**	**Adjusted OR (95% CI)**^**a,b**^
Age 18–24	1.00	1.00
Age 25–34	0.76 (0.59–0.97)*	0.93 (0.65–1.32)
Age 35–44	0.77 (0.60–1.00)	1.00 (0.68–1.45)
Age 45 and older	0.80 (0.61–1.05)	0.65 (0.43–1.00)
Female	1.00	1.00
Male	1.26 (1.07–1.48)**	1.09 (0.86–1.38)
Grade 7 or less	1.00	1.00
Grade 8–11	0.88 (0.72–1.06)	1.07 (0.80–1.43)
Grade 12 or more	0.68 (0.55–0.85)***	0.76 (0.54–1.07)
Poverty low	1.00	1.00
Poverty medium	1.97 (1.62–2.410***	1.73 (1.34–2.24)***
Poverty high	4.01 (3.12–5.10)***	1.65 (1.14–2.39)**
Health variables		
Perceived health status		
Excellent/Very good	1.00	1.00
Good	0.36 (0.29–0.45)***	0.50 (0.37–0.67)***
Fair/Poor	0.24 (0.20–0.30)***	0.44 (0.32–0.60)***
TB retreatment patient	1.00	–
New TB patient	0.82 (0.67–1.00)	
HIV positive	1.00	1.00
HIV negative	0.40 (0.34–0.48)***	0.44 (0.33–0.59)***
Chronic conditions		
Zero	1.00	1.00
One	1.54 (1.23–1.93)***	1.86 (1.41–2.46)***
Two	1.84 (1.34–2.51)***	2.44 (1.68–3.56)***
Three or more	1.98 (1.34–2.92)***	2.37 (1.45–3.88)***
Severe psychological distress (K ≥ 30)	1.31 (1.09–1.57)**	0.94 (0.73–1.22)
Alcohol: low risk (AUDIT 0–7)	1.00	1.00
Medium (AUDIT 8–19)	1.74 (1.42–2.19)***	1.65 (1.23–2.29)***
High (AUDIT 20–40)	2.42 (1.76–3.32)***	3.06 (1.94–4.81***
Current tobacco use	2.09 (1.76–2.49)***	1.35 (1.04–1.75)*
On ART	0.70 (0.56–0.86)***	0.78 (0.60–1.03)
Partner HIV negative/unknown	1.00	1.00
Partner HIV positive	1.41 (1.16–1.70)***	1.43 (1.10–1.84)**

*P < 0.05; ** P < 0.01; *** P < 0.001.

^a^Using “enter” LR selection of variables.

^b^Hosmer and Lemeshow Chi-square 7.68, df8, 0.466; Cox and Snell R^2^ 0.11; Nagelkerke R^2^ 0.16.

Univariate analysis (see Table 2) shows that the following factors were more likely to be associated with adherence to TB treatment: being in the 25–34 year age group [OR: 0.76 (0.59–0.97), p < 0.05], having a grade12 or more education [OR: 0.68 (0.55–0.85), p < 0.001], perceiving health status as being ‘poor’ [OR: 0.24 (0.20–0.30), p < 0.001], perceiving health status to be ‘good’ [OR: 0.36 (0.29–0.45), p < 0.001], being HIV negative [OR: 0.40 (0.34–0.48), p < 0.001], and being on ART [OR: 0.70 (0.56–0.86), p < 0.001].

Multivariable analysis (see Table 2) indicates that the following factors are more likely to predict TB treatment non-adherence: medium levels of poverty [OR: 1.73 (1.34–2.24), p < 0.001], high levels of poverty [OR: 1.65 (1.14–2.39), having one (1) chronic condition [OR: 1.86 (1.41–2.46), p < 0.001], having two (2) chronic conditions [OR: 2.44 (1.68–3.56), p < 0.001], having three or more chronic conditions [OR: 2.37 (1.45–3.88), p < 0.001], medium risk for alcohol misuse [OR: 1.65 (1.23–2.29), p < 0.001], high risk for alcohol misuse [OR: 3.06 (1.94–4.81), p < 0.001], tobacco use [OR: 1.35 (1.04–1.75), p < 0.05], and having a partner who is HIV positive [OR: 1.43 (1.10–1.84), p < 0.01].

Multivariable analysis (see Table 2) indicates that the following factors are more likely to predict adherence to TB treatment: perceiving health status as being ‘poor’ [OR: 0.44 (0.32–0.60), p < 0.001], perceiving health status to be ‘good’ [OR: 0.50 (0.37–0.67), p < 0.001], and being HIV negative [OR: 0.44 (0.33–0.59), p < 0.001].

### Predictors of non-adherence to dual therapy (anti-TB treatment and ART)

Univariate analysis (see Table 3) indicates that the variables significantly associated with non-adherence to dual therapy were: being male [OR: 1.61 (1.20–2.16), p < 0.001], being in the medium poverty category [OR: 3.14 (2.05–4.79), p < 0.001], being in the high levels of poverty category [OR: 7.13 (4.36–11.66)], having three or more chronic conditions [OR: 2.45 (1.33–4.5), p < 0.01], medium risk for alcohol misuse [OR: 1.65 (1.11–2.45), p < 0.05], high risk for alcohol misuse [OR: 2.76 (1.30–5.86), p < 0.01], tobacco use [OR: 2.30 (1.65–3.19), p < 0.001], having a partner who is HIV positive [OR: 2.41 (1.75–3.32), p < 0.01], and having sex in the past three months [OR: 1.44 (1.07–1.93), p < 0.05].

**Table 3:** Predictors of non-adherence to ART and anti-TB medication (N = 757)

**Socio-demographics**	**Crude OR (95% CI)**	**Adjusted OR (95% CI)**^**a,b**^
Age 18–34	1.00	1.00
Age 35–44	0.68 (0.49–0.95)*	0.63 (0.38–1.03)
Age 45 and older	1.12 (0.76–1.64)	0.90 (0.49–1.67)
Female	1.00	1.00
Male	1.61 (1.20–2.16)***	1.49 (0.95–2.32)
Grade 7 or less	1.00	1.00
Grade 8–11	0.66 (0.47–0.93)*	1.13 (0.67–1.93)
Grade 12 or more	0.79 (0.53–1.17)	1.19 (0.65–2.18)
Poverty low	1.00	1.00
Poverty medium	3.14 (2.05–4.79)***	2.60 (1.46–4.65)***
Poverty high	7.13 (4.36–11.66)***	3.89 (1.87–8.12)***
Health variables		
Perceived health status		
Very good/good	1.00	1.00
Good	0.41 (0.28–0.59)***	0.69 (0.40–1.17)
Fair/Poor	0.20 (0.14–0.29)***	0.28 (0.17–0.49)***
TB retreatment patient	1.00	1.00
New TB patient	0.51 (0.37–0.71)***	0.67 (0.42–1.07)
Chronic conditions		
Zero	1.00	1.00
One	1.39 (0.91–2.12)	1.72 (1.01–2.94)*
Two	1.50 (0.87–2.58)	2.73 (1.31–5.65)**
Three or more	2.45 (1.33–0.45)**	5.33 (2.27–12.55)***
Severe psychological distress (K ≥ 30)	1.00 (0.72–1.37)	–
Alcohol: low risk (AUDIT 0–7)	1.00	1.00
Medium (AUDIT 8–19)	1.65 (1.11–2.45)*	1.08 (0.56–2.09)
High (AUDIT 20–40)	2.76 (1.30–5.86)**	13.09 (2.96–57.99)*
Current tobacco use	2.30 (1.65–3.19)***	1.44 (0.85–2.43)
Partner HIV negative/unknown	1.00	1.00
Partner HIV positive	2.41 (1.75–3.32)***	3.12 (1.84–5.29)***
Had sex in past 3 months	1.44 (1.07–1.93)*	1.48 (0.96–2.28)
Sex partner on ART	0.51 (0.35–0.74)***	0.50 (0.25–0.97)*

*P < 0.05; ** P < 0.01; *** P < 0.001.

^a^Using “enter” LR selection of variables.

^b^Hosmer and Lemeshow Chi-square 13.14df8, 0.107; Cox and Snell R^2^ 0.24; Nagelkerke R^2^ 0.33.

The factors that are more likely to be associated with adherence to dual therapy as indicated by the univariate analysis are: being in the 35–44 year age group [OR: 0.68 (0.49–0.95), p < 0.05], having a grade 8 to 11 education [OR: 0.66 (0.47–0.93), p < 0.05], perceiving health status as being ‘poor’ [OR: 0.20 (0.14–0.29), p < 0.001], perceiving health status to be ‘good’ [OR: 0.41 (0.28–0.59), p < 0.001], being a new TB patient [OR: 0.51 (0.37–0.71), p < 0.001], and having a sex partner on ART [OR: 0.51 (0.35–0.74), p < 0.001].

The results of the multivariate analysis as indicated in Table 3 highlights the following significant predictive factors for non-adherence to dual therapy in the sub-sample of TB patients co-infected with HIV were: medium poverty [OR: 2.60 (1.46–4.65), p < 0.001], high levels of poverty [OR: 3.89 (1.87–8.12), p < 0.001], having one (1) chronic condition [OR: 2.73 (1.31–5.65), p < 0.01], having three (3) or more chronic conditions [OR: 5.33 (2.27–12.55), p < 0.001], high risk for alcohol misuse [OR: 13.09 (2.96–57.990), p < 0.05] and having a partner who is HIV positive [OR: 3.12 (1.84–5.29), p < 0.001].

Finally, the following predictive factors were associated with adherence to dual therapy: perceiving health status as being ‘poor’ [OR: 0.20 (0.14–0.29), p < 0.001] and having a sex partner on ART [OR: 0.50 (0.25–0.97), p < 0.05].

## Discussion

The sub-sample of HIV positive patients on dual therapy for TB and HIV infection, in this study, shared a similar economic and alcohol misuse profile to the total sample of TB infected patients. In addition, nearly 40% of patients on dual therapy reported having a partner who is HIV positive. Finally, the self-report of non-adherence to dual therapy is high in this sub-sample (42.4%) but comparable to the figures reported in the literature [2]. Non-adherence to both ART and anti-TB drugs are of concern in this sub-group. However, given the fact that this sub-group has a dual infection requires that they take two sets of drugs, which could prove to be quite burdensome. The use of a multiple drug regimen may also lead to drug reactions causing unmanageable side-effects ultimately leading to treatment default [28-30]. The complexity of the drug treatment regimen and the impact on the daily lives of these patients are also factors that are associated with poor adherence in these patients [29,30]. Patients on strict drug treatment programmes also have daily competing “life demands” associated with work and family.

The four common predictive factors independently associated with non-adherence to anti-TB drugs and to dual therapy (i.e. ART and anti-TB drugs) were poverty, having co-morbid disease conditions, being at risk for alcohol misuse and having a HIV positive partner. This finding is supported by existing literature on the relationship between the social determinants of health and health outcome and/or the quality of life of individuals with one or more disease condition [31,32]. In this study non-adherent behavior for both anti-TB drugs and ART was associated with a lack of economic resources (poverty) and negative personal circumstances (co-morbid conditions and a HIV positive partner). Clearly the lack of social, personal and economic resources is a barrier to adherence. Unfortunately, the lack of resources, in particular poverty, has historically been associated with TB onset [33]. It stands to reason, therefore, that being poor is a barrier to health promoting behavior, such as adhering to a treatment programme, because individuals faced with economic restraints do not have an enabling environment that facilitates behavior that will lead to better health outcomes. Being poor, having negative personal circumstances and engaging in risky behavior such as alcohol misuse is likely to lead to poor health outcomes as a consequence of treatment default [13,34]. It is plausible that patient mis-use of alcohol, in particular, may have led to poor and impaired judgement [35,36] when making health decisions, such as not adhering to their anti-TB medication**.**

Of particular concern in this study is the fact that HIV negative participants’ who were non-adherent to anti-TB drugs and in a relationship with a HIV positive partner, placed not only themselves but their partners at risk for poor health outcomes. Inconsistent adherence to anti-TB drugs may not only lead to MDR and XDR-TB but may increase the risk of transmission of TB to others living and working in close proximity. The HIV positive patients, in this study, who were not adhering to dual therapy, placed themselves at risk for drug resistance which could in turn reduce their life-span. ART, taken in accordance with treatment guidelines is known to prolong an HIV infected person’s life and the availability of ART has led health professionals to re-conceptualize HIV and AIDS as chronic medical conditions.

One additional factor, namely tobacco use, was independently associated with non-adherence to TB treatment drugs. Individuals who smoke tobacco may be conceived of as having a personality with an “increased risk profile” which makes them prone to engaging in undesirable behaviours regardless of the consequences [35,36]. It is not surprising, therefore, that participant’s adherence to anti-TB drugs in this study, was mediated by tobacco smoking.

The common predictive factor independently associated with adherence to anti-TB drugs and to dual therapy (i.e. ART and ant-TB drugs) was: perceiving health status to be poor. This finding may seem counter-intuitive at first glance. It is reasonable, however, that the participants who perceived that their health status was poor on the basis that they had a “double-disease” burden of being infected with TB and HIV were more motivated to take the medication which they know to be an effective form of treatment. Knowledge of medical treatment efficacy for a specific disease condition is known to influence the adherence behavior of the recipients of care [5].

Additional predictive factors for adherence to TB treatment were: perceiving health status to be good and being HIV negative. This finding is supported by the literature that reports on factors found to be influencing adherence to medical treatment regimens for communicable and non-communicable diseases. Individuals with a transient medical condition, that can be cured, equipped with the knowledge that heeding to health care practitioner’s suggested treatment regimens for cure are more likely to adhere [37]. Of course there are numerous other factors that may influence adherence to medication for a curable disease, such as TB. These factors include knowing someone who was cured, the need to maintain a good health status, and having a high level of health literacy (such as knowledge of disease transmission as in the case of TB) [7,32].

The results indicate that there is a trend between perceived health status and adherence in the anti-TB treatment group and the dual treatment group. In the anti-TB treatment group health status perceived to be good is associated with adherence to treatment but this is not the case in the smaller group receiving dual treatment.

Finally, the additional predictive factor for adherence to dual therapy (i.e. anti-TB treatment and ART) was having a sex partner on ART. Having a sex partner on ART is in some way a protective factor for an individual who is also on ART because of the sero-concordant nature of the relationship. Both partners in this relationship are able to support each other in an open manner without fear of being stigmatized for being HIV positive [38,39]. Non-disclosure of certain medical conditions, such as having HIV or AIDS, is known to fuel the epidemic [40]. Consequently, one may assume that in households where there is non-disclosure of HIV status, the adherence to ART will be non-existent, poor or inconsistent for fear of one’s HIV positive status being revealed [41]. In essence social support has a positive association with medical treatment adherence [42].

## Conclusion

A major limitation of this study is that it is cross-sectional. Ideally, the adherence patterns of TB patients and those dually infected with HIV should be tracked over time in order to better understand how the disease course(s) influences the patient’s willingness to co-operate with the prescribed treatment. Despite this limitation, the results of this study provide insight into the factors that are associated with non-adherence to treatment among TB patients and those co-infected with HIV. Given the factors found to be associated with non-adherence in this study, a comprehensive treatment programme using a patient-centred model is indicated for TB patients attending public health care clinics in SA [5]. This comprehensive treatment programme being advocated should address structural factors (such as poverty); risky behaviours (such as alcohol mis-use and tobacco use); and psycho-social well-being (such as social relationship counseling on engaging with a sero-discordant partner is indicated for TB patients [with and without HIV]) and counseling related to perceived health status and living with co-morbid health conditions). The treatment care package needs to involve not only the health sector but other relevant government sectors, such as social development.

## Competing interests

The authors declare that they have no competing interests.

## Authors’ contributions

PN and KP were primarily responsible for the conceptualization of the study, for strategic guidance on the project and the first draft of this manuscript. They were assisted by GM during the grant application phase and application for ethical approval of the study. JL, GM, GM and BT were involved in the operational aspects of the research process. All authors read and approved the final manuscript.

## Pre-publication history

The pre-publication history for this paper can be accessed here:

http://www.biomedcentral.com/1471-2458/13/396/prepub
